# Adverse Effects Faced by Healthcare Workers While Using Personal Protective Equipment During the COVID-19 Pandemic in Civil Hospital, Ahmedabad

**DOI:** 10.7759/cureus.38485

**Published:** 2023-05-03

**Authors:** Rosy Laxmidhar, Chetna Desai, Prakruti Patel, Fehmida Laxmidhar

**Affiliations:** 1 Infectious Diseases, Byramjee Jeejeebhoy (BJ) Medical College, Civil Hospital Asarwa, Ahmedabad, IND; 2 Pharmacology, Byramjee Jeejeebhoy (BJ) Medical College, Civil Hospital Asarwa, Ahmedabad, IND; 3 Infectious Diseases, Smt. Nathiba Hargovandas Lakhmichand (NHL) Municipal Medical College, Ahmedabad, IND

**Keywords:** covid-19, healthcare worker, skin involvement, pandemic challenges, ppe, adverse effects, personal protective equipment

## Abstract

Background

Healthcare workers (HCWs) were compelled to use personal protective equipment (PPE) during the COVID-19 pandemic to prevent cross-transmission. One of the most significant challenges in responding to the COVID-19 pandemic is the consistent and effective use of PPE to avoid staff exposure and infection. This study aimed to detect and evaluate the adverse effects of PPE and determine the associated risk factors.

Methodology

This cross-sectional study included 186 randomly selected HCWs at Civil Hospital, Ahmedabad, from May 2022 to July 2022. An anonymous self-administered questionnaire was used for data collection, and data analysis was done using descriptive statistics.

Results

PPE-related adverse effects were noted among 147 HCWs, with a prevalence of 79.03%. Data analysis showed that factors significantly associated with PPE adverse effects in HCWs were age group 20-40 years (chi-squared (χ^2^) = 4.119, p = 0.04) and female gender (χ^2^ = 7.153, p = 0.007). Overall, 30.8% of participants had tested positive while on duty during the pandemic. Similarly, adverse effects were associated with PPE use of more than four hours per day and more than three days per week (χ^2^ = 5.477, p = 0.02 and χ^2^ = 6.488, p = 0.01, respectively). The majority of HCWs expressed indentation and pain on the back of the ear (52.7%) and pressure-related injury (39.8%) as adverse effects after wearing masks; skin soaking in sweat (54.83%) due to gloves; profuse sweating due to gown (64.28%); fogging (65.26%) due to googles and face-shield; and discomfort (61.29%).

Conclusions

The prevalence of adverse effects related to wearing PPE was alarmingly high among HCWs. The major risk factors were age, female sex, and duration of use. Although the majority of healthcare personnel have received vaccinations, the use of PPE has not altered, and severe skin reactions continue to be a global issue with no known solution. To further understand the problem, national data for the impacted healthcare professionals could be helpful. Furthermore, workplace prevention programs are necessary.

## Introduction

Personal protective equipment (PPE) is utilized to prevent or reduce occupational dangers and unexpected workplace accidents. Gloves, gowns, shoe covers, masks, face shields, and goggles are components of PPE.

In late December 2019, reports of severe acute respiratory illnesses emerged from Wuhan in Hubei Province, China. By January 2020, the disease now known as coronavirus disease 2019 (COVID-19), caused by the severe acute respiratory syndrome coronavirus 2 (SARS-CoV-2), had quickly spread from Wuhan to neighboring areas [1]. The World Health Organization (WHO) declared COVID-19 a pandemic on March 11, 2020. India recorded 44,500,580 COVID-19 cases, with 528,165 deaths attributed to the disease as of August 2022.

In response, healthcare workers (HCWs) have been mobilized to treat patients with COVID-19. The Centers for Disease Control and Prevention (CDC) and WHO recommend adopting standard precaution measures, including gowns, gloves, and eye protection. During the COVID-19 pandemic, PPE was compulsory for frontline HCWs in high-risk areas, such as isolation wards, emergency rooms (equipped with a fever facility), and the medical intensive care unit (MICU). Healthcare professionals are more susceptible to infection during outbreaks of highly contagious diseases such as Ebola or SARS than the general public [2]. Because the respiratory tract is the main transmission route of the virus, HCWs must use PPE constantly [3].

It has been exceedingly difficult to manage the COVID-19 pandemic effectively and consistently as using PPE has adverse physical and psychological effects on HCWs and patients. As the outbreak escalated, it was necessary for HCWs to rapidly develop skills in the proper wearing of PPE to ensure personal protection from exposure to COVID-19. Changes to PPE policy and practices had to be made as the pandemic progressed and our knowledge of viral transmission mechanisms improved. This dynamic policy environment also made consistent training of HCWs in optimal PPE use challenging [4].

The excessive and prolonged use of PPE is associated with many adverse effects ranging from headaches, pressure injuries, dehydration, itching, erythema, and acne to decreased work efficiency [1,2]. Various risk factors impact the severity of adverse effects, for example, age, gender, duration of work, environmental temperature, and skin sensitivity to the PPE material [5]. There is a need to understand these problems faced by HCWs so that they can take preventive steps; the manufacturers can modify the material of PPE or, if need be, withdraw inferior quality PPE from the market. Although studies have begun to examine and expose the problems associated with PPE use globally, India still needs more authentic research on the subject [6]. Several recent articles have highlighted concerns regarding the negative impacts of PPE use. Such arguments, however, could be more persuasive in the presence of essential replicable data. The data collected can help HCWs and administrators to make informed decisions while forming clinical health policies.

## Materials and methods

This study was designed as a cross-sectional study conducted at Civil Hospital, a tertiary referral center in Ahmedabad. The study was conducted from May 2022 to July 2022. The research method used was a questionnaire in the form of a Google Form, which was shared through various social media platforms (Table 1). The questionnaire was developed after a thorough literature review and discussion with healthcare professionals about the adverse effects experienced by them. The questionnaire consisted of multiple-choice questions with mandatory fields marked. Some questions allowed participants to select multiple options and provide additional symptoms they may have experienced. The first part of the questionnaire collected demographic (gender, age) and professional data, while the second part focused on underlying skin conditions, PPE usage patterns (type of mask and gloves used, average number of hours of each piece of equipment used, and number of days per week of PPE usage), and adverse effects of different components of PPE. Lastly, information was collected on whether they had tested positive for COVID-19 while on duty.

**Table 1:** An outline of the questionnaire used in the study. PPE: personal protective equipment; COVID-19: coronavirus disease 2019

Questionnaire
Consent of participant:
I give my permission to record my responses to this questionnaire. I understand that their work is for research purposes. The research title is “Adverse Effects Faced by the Healthcare Workers While Using Personal Protective Equipment During the COVID-19 Pandemic in Civil Hospital, Ahmedabad.” I also understand that the researcher will maintain my anonymity about my responses to questionnaire items.
(Please tick the relevant option):
a) I agree to give my consent
b) I refuse to give my consent
1. Name (initials only): ________________
2. Occupation:
a) Doctor
b) Nurse
c) Others______
3. Gender: _______
4. Age:
a) 20 to 40 years
b) 41 to 60 years
5. Did you use PPE during your COVID-19 duty?
a) Yes
b) No
6. Number of days of wearing PPE?
a) Once a week
b) 2–3 days per week
c) 4–5 days per week
d) Whole week
e) Specify duration: ______
7. Parts included in PPE:
a) Mask
b) Gloves
c) Gown and shoe cover
d) Goggles
e) Face shield
8. What type of mask was used?
a) N95
b) Surgical
c) Cloth
d) Others:
9. Average number of hours per day of wearing the mask?
a) Less than or equal to 4 hours
b) More than 4 hours
10. Any underlying skin conditions?
a) Yes (please specify) ______
b) No
11. Skin problems after mask use:
a) Red rash, rosacea, pigmentation
b) Acne
c) Indentation and pain in the back of the ears
d) Perioral dermatitis
e) Itching
f) Pressure-related skin injury (e.g., nasal bridge scar)
g) None of the above
h) Others: _____
12. Did you use gloves?
a) Yes
b) No
13. What type of gloves?
a) Latex
b) Latex free
c) Others (please specify): _______
14. Duration (per day) of wearing gloves?
a) Less than or equal to 4 hours
b) More than 4 hours
15. Skin problems after the use of gloves:
a) Dryness
b) Hand erythema and itching
c) Vesicles
d) Skin soaking in sweat
e) None
f) Others (please specify): _____
16. Did you use the gown of PPE?
a) Yes
b) No
17. Duration (per day) of wearing gown:
a) Less than or equal to 4 hours
b) More than four hours
18. Problems faced during the use of gown:
a) Erythema on the body
b) Profuse sweating
c) Pruritus
d) Dry skin
e) Tearing of gown
f) Soaking of a gown with blood and biological fluids
g) None
h) Others (please specify): _____
19. Did you use goggles?
a) Yes
b) No
20. Duration (per day) of use of goggles and face shield:
a) Less than or equal to 4 hours
b) More than four hours
21. Side effects of using goggles:
a) Pressure-related skin injury
b) Fogging
c) Decreased visibility
d) None
e) Others (please specify): ________
22. Did you use a face shield?
a) Yes
b) No
23. Duration per day of face shield use:
a) Less than or equal to 4 hours
b) More than four hours
24. Side effects of using a face shield:
a) Pressure-related skin injury
b) Fogging
c) Decreased visibility
d) None
e) Others (please specify): _______
25. Did you experience any of the following?
a) Dizziness
b) Discomfort
c) Fatigue
d) Appropriate size not available
26. Have you tested positive for COVID-19 during your duty?
a) Yes
b) No
27. If yes, when? _______

The subject selection for this study employed a grab sampling or availability sampling approach. All HCWs who used PPE during the COVID-19 pandemic while performing their duties at Civil Hospital, Ahmedabad, including doctors, nurses, support staff, laboratory assistants, laboratory technicians, medical students, and sanitation workers, were invited to participate in the study. HCWs who used PPE during the pandemic but refused to participate in this study were excluded. For data collection, an online survey was sent using Google Forms from May 2022 to July 2022 after obtaining approval from the Institutional Ethics Committee B.J. Medical College and Civil Hospital, Ahmedabad (approval number: 114/2022). Participants who were not comfortable completing the online survey were offered a hard copy of the questionnaire instead. The questionnaire was voluntary, and responses were kept confidential. Participants were asked to consent before responding and were informed that the data collected by the questionnaire would be solely used for research purposes. Participants were given reminders at regular intervals during the study period to ensure a high response rate.

Statistical data analysis was performed using Microsoft Excel after collecting the primary data. The chi-square test was employed to identify variables associated with the prevalence of unfavorable effects of PPE use among HCWs. Specifically, we analyzed the relationship between adverse effects and variables such as demographic characteristics, underlying skin conditions, PPE usage patterns, and the different components of PPE. Statistical significance was defined as a p-value of 0.05.

## Results

The survey tool was sent through social media to about 400 HCWs, and valid responses were obtained from 186 HCWs (46.5% response rate).

The baseline characteristics of the population under study are summarized in Table 2. The number of HCWs using different components of PPE and the prevalence of adverse effects are shown in Table 3. The participants were split into two age groups; 150 participants were aged between 20 and 40 years (80.60%), and 36 were aged between 41 and 60 years (19.30%). In total, there were 124 (66.6%) females, and the rest were males (33.3%). There were 75 (40.3%) doctors, 59 (31.72%) nurses, and 52 (27.95%) allied HCWs which included laboratory technicians, laboratory assistants, sweepers, research scientists, support staff, and medical students. In total, 12 (6.45%) participants reported underlying skin diseases, such as allergic dermatitis and acne. Overall, 147 (79%) participants reported that wearing PPE had one or more negative consequences.

**Table 2:** Baseline characteristics of the population under study. HCWs: healthcare workers; PPE: personal protective equipment

Characteristics	Number	Percentage
Gender		
Male	62	33.30%
Female	124	66.60%
Age category		
20–40 years	150	80.60%
41–60 years	36	19.30%
Profession		
Doctor	75	40.30%
Nurse	59	31.72%
Allied HCWs	52	27.95%
People with underlying skin disease	12	6.45%
Types of PPE used		
Masks (n = 186)		
N95	179	96.00%
Surgical	7	3.76%
Gloves (n = 186)		
Latex	164	88.20%
Latex free	22	11.80%
Gowns	182	97.84%
Goggles and face shield	167	89.78%
Duration of PPE use per week		
3 days	47	25.26%
>3 days	139	74.73%
Duration of PPE use per day(hours)		
4 hours	45	24.19%
>4 hours	141	75.80%
Side effects related to PPE	147	79.03%

**Table 3:** Number of HCWs using different components of PPE and prevalence of adverse effects. HCWs: healthcare workers; PPE: personal protective equipment; A/E: adverse effects

Type of PPE	A/E present	A/E absent
Masks (n = 186)		
N95	142 (79.32%)	37 (20.67%)
Surgical	5 (71.42%)	2 (28.57%)
Gloves (n = 186)		
Latex	133 (81.09%)	31 (18.9%)
Latex free	18 (81%)	4 (18.18%)
Gowns (n = 182)	138 (75.82%)	44 (24.17%)
Goggles and face shield (n = 167)	130 (77.84%)	37 (22.15%)

The main adverse events related to the wearing of PPE among healthcare workers are presented in Table 4. The main adverse effects of mask-wearing were indentation and pain on the back of the ear in 98 (52.7%) HCWs, pressure-related skin injury (e.g., nasal bridge scar) in 74 (39.8%) HCWs, and itching in 51 (27.4%) HCWs. The main adverse effects of gloves were skin soaking in sweat in 102 (54.8%) HCWs and dryness in 72 (38.7%) HCWs. Fogging in 109 (65.26%) HCWs and profuse sweating in 117 (64.28%) HCWs were the significant adverse effects of gowns and goggles/face shields, respectively. Wearing all the components of PPE led to discomfort in 114 (61.29%) HCWs.

**Table 4:** Main adverse events related to the wearing of PPE among HCWs. HCWs: healthcare workers; PPE: personal protective equipment; A/E: adverse effects

PPE	Main related A/E	Number	Percentage
Masks (n = 186)	Red rash, rosacea, pigmentation	37	19.90%
	Acne	29	15.60%
	Indentation and pain in the back of the ear	98	52.70%
	Perioral dermatitis	8	4.30%
	Itching	51	27.40%
	Pressure-related skin injury (e.g., nasal bridge scar)	74	39.80%
	None	39	21%
Gloves (n = 186)	Dryness	72	38.70%
	Hand erythema and itching	40	21.50%
	Vesicles	9	4.83%
	Skin soaking in sweat	102	54.83%
	None	35	18.81%
Gown (n = 182)	Erythema on body	6	3.30%
	Profuse sweating	117	64.28%
	Pruritis	16	8.80%
	Dry skin	42	23.07%
	Tearing of gown	31	17%
	Soaking the gown with body fluids and blood	9	4.90%
	None	44	24.17%
Goggles and face shield (n = 167)	Pressure-related skin injury	14	8.38%
	Fogging	109	65.26%
	Decreased visibility	54	32.33%
	None	37	22.15%
Physical health problems	Dizziness	40	21.50%
	Discomfort	114	61.29%
	Fatigue	66	35.40%
	Appropriate size not available	33	17.70%

Factors linked to negative PPE-related occurrences among healthcare professionals are presented in Table 5. In the univariate analysis, sociodemographic factors that were statistically associated with PPE-related adverse effects among HCWs were age group 20-40 years (chi-squared (χ^2^) = 4.119, p = 0.04) and female sex (χ^2^ = 7.153, p = 0.007). More than four hours of PPE usage daily and more than three days per week were significantly linked to negative impacts (χ^2^ = 5.477, p = 0.02 and χ^2^ = 6.488, p = 0.01, respectively).

**Table 5:** Factors linked to negative PPE-related occurrences among healthcare professionals. A/E: adverse events; PPE: personal protective equipment; *: significant value p <0.05

Variables	A/E present	A/E absent	Chi-square value	P-value
Age group				
20–40 years	123 (82%)	27 (18%)	4.119	0.04*
41–60 years	24 (66.6%)	12 (33.4%)		
Gender				
Male	42 (67.7%)	20 (32.25%)	7.153	0.007*
Female	105 (84.67%)	19 (15.32%)		
Profession				
Doctor	63 (84%)	12 (16%)	3.403	0.182
Nurse	42 (71.18%)	17 (28.8%)		
Allied HCWs	42 (80.76%)	10 (19.2%)		
Duration of PPE use per day				
4 hours	30 (66.66%)	15 (33.33%)	5.477	0.02^*^
>4 hours	117 (82.26%)	24 (17.02%)		
Duration of PPE use per week				
≤3 days	31 (65.95%)	16 (34.04%)	6.488	0.01*
>3 days	116 (83.45%)	23 (16.54%)		
Type of PPE				
Masks				
N95	142 (79.32%)	37 (20.67%)	0.001	0.974
Surgical	5 (71.42%)	2 (28.57%)		
Gloves				
Latex	133 (81.09%)	31 (18.9%)	0.044	0.833
Latex free	18 (81%)	4 (18.18%)		

Of the total participants, 128 (69%) did not test positive for COVID-19 while on duty during the pandemic, while 58 (31%) tested positive.

## Discussion

Along with other measures protecting against infectious agents, rational use of PPE can ensure less risk of infection among HCWs. In this study, the prevalence of PPE-related adverse effects was noted to be 79.03%, which can be compared to previous similar studies conducted in other countries (75%, 80%, 78%) [7-9]. Studies conducted in southern Tunisia [10] and Singapore [4] reported a lower prevalence of these adverse effects (52.3% and 53.8%, respectively). The methodological approach used by different studies can account for this variation. During the COVID-19 pandemic, HCWs from other nations also reported a high prevalence of PPE-related side effects, with up to 97% of HCWs in China experiencing skin reactions after continuous PPE use [9,11].

Furthermore, this study demonstrated that female HCWs most commonly reported PPE-associated adverse effects, similar to previous studies showing this gender discrepancy [4,12,13]. This result may be explained by the fact that males tend to pay less attention to skin reactions caused by PPE wear than women and the different standards between the sexes regarding how they perceive, express, and report discomfort or other negative impacts. This result may also be due to gender disparities in cosmetic use and work habits, such as the employment of women in occupations requiring more extended PPE use. Some studies also reported that men are less likely to wear masks and other components of PPE, which might also result in fewer reports of adverse effects [12]. In this study, adverse effects were seen in those who reported previous underlying skin diseases, which may contribute to the skin’s fragility. The older age group (41-60 years) was less likely to report adverse effects, which may be due to differences in the onset of acne and other skin disorders associated with age. This information backs up the conclusions of a prior study from Singapore [14] conducted during the SARS pandemic. Long periods spent wearing PPE resulted in skin friction and discomfort, excessive sweating, and a heated environment that encouraged microbial growth on the skin. If HCWs utilized PPE for an extended period, these two circumstances were conducive to developing unfavorable consequences [10]. Similarly, other studies revealed that using PPE for an extended period increases the likelihood of adverse outcomes [4,7,10,13].

Masks were an essential component of PPE to prevent the transmission of COVID-19. The CDC and WHO recommended N95 masks for HCWs working during the COVID-19 pandemic. Their prolonged use resulted in indentation and pain in the back of the ear and pressure-related skin injury (e.g., nasal bridge scar) as the significant adverse effect. Similar results were observed in a study in the Chinese province of Hubei [15]. For every degree Celsius that the temperature of the mask-covered face rises, the rate at which sebum is excreted increases by 10%. This encourages the development of acne-like lesions in people who already have excessive sebum production, especially when combined with the usage of surgical masks. Rash, acne, and pigmentation were the most prevalent side effects in the study by Foo et al. [14]. HCWs who tie the N95 respirator masks tightly and squeeze the metal strip firmly to guarantee tightness for the best infection protection likely experience these skin reactions. Due to hypoxemia, this activity causes physical issues such as headaches and vertigo.

All 186 responders said they regularly wore gloves and had unpleasant hand-skin reactions. The most frequently reported adverse effects of glove use were skin soaking in sweat and dry hands. High environmental temperatures and tight gloves caused excessive sweating and skin chafing. It is possible to prevent allergic dermatitis by thoroughly drying hands before putting on gloves and by putting plastic gloves inside latex gloves [16]. No statistically significant correlation between the kind of glove material and the occurrence of any symptom (such as dryness, itching, and abrasion) was noted on the chi-square tests of association. This suggests that skin irritation, particularly on the hands, is more likely to be caused by usage rather than an allergic reaction to a specific material. This study showed no difference in the number of adverse effects due to latex and latex-free gloves, which contradicts the well-established correlation between increased use of latex gloves and skin problems [12,13]. A possible reason is the smaller sample size of people wearing latex-free gloves. Further research is necessary to clarify this finding. Additionally, exploring the precise physiological etiology of adverse skin reactions related to PPE would be a valuable topic for future studies.

The adverse effects of gowns were comparatively fewer, and profuse sweating was the most frequently reported adverse effect. These findings were congruent with previous studies [4,17]. This study was conducted during the summer season, with temperatures rising to about 44°C, which is likely the reason for the profuse sweating. Consistent with reports from Jose et al. [13], HCWs reported fogging of goggles and face shields, which reduced their visibility and hindered their work. The issue may have been made worse by the humidity in the air and the absence of air conditioning in some workplaces. The anti-fogging capabilities of goggles and face shields can be improved in several ways, but further research is required to determine the most efficient solutions. Applying cleaning or anti-mist agents to goggles or glasses may be beneficial temporary solutions.

Most HCWs experience xerosis, pruritus, erythema, papules, and maceration due to PPE usage for an extended period, according to early data from China and other publications. An Italian survey among 1223 HCWs reported that these negative consequences are a developing occupational health concern. Ninety medical monitoring visits were required due to dermatological issues connected to PPE [12].

There is a dearth of clinical information about skin reactions to PPE use, and there is a great deal of uncertainty about when PPE, particularly face masks, should be used in public and clinical settings. Since the start of COVID-19, the CDC has published various revisions to its PPE guidelines. Despite recent stabilization, they still do not offer recommendations for the daily length of PPE use or for reducing adverse reactions among HCWs. Therefore, observational studies may contribute to the production of data that could guide the creation of such regulations in the future.

All adverse effects of PPE are time-dependent. A well-described risk factor for various inflammatory lesions is the prolonged use of these devices, which promotes the development of a warm, moist, occlusive environment. The main risk factors identified in this study for developing cutaneous adverse effects related to PPEs are previous dermatological diseases, utilization duration, and female sex. Appropriate measures must be taken to minimize the time of PPE use by HCWs. These include taking a 15-minute break from the mask every two hours, wearing N95 mask straps on the crown to reduce pressure on the ears, covering places where PPE is constantly in touch with alcohol-free film barriers, drying hands thoroughly before donning gloves, and applying petrolatum to damaged skin [18,19]. Training programs should be conducted to ensure proper donning and doffing of PPE. As long working hours are related to a higher risk of adverse effects, they should be regulated. The temperatures of the working environment should be controlled to ensure maximum comfort while wearing PPE [20]. The inability to address PPE-related problems may lead to absenteeism and refusals to work in COVID-19 units.

This cross-sectional study is susceptible to bias similar to all survey-based studies. Non-response bias is the most probable cause of bias in this study. People with negative experiences are more likely to complete the survey, which can skew the results in favor of more significant estimates of the prevalence and severity of skin responses. Regular reminders were sent to HCWs to fill out the questionnaire to minimize this bias, and simple, single-sentence questions were asked. Short, direct survey items were designed in a multiple-choice format for easy answering. No incentive was given for the completion of the survey.

However, these results may have some limitations. Data regarding the adverse effects of PPE were collected only from HCWs at Civil Hospital Ahmedabad, a tertiary institution, so it may only be possible to generalize the findings to some healthcare settings. Steps taken by individual participants to curb these adverse effects, such as bleach, chemical cosmetics, or other disinfection products, were not taken into consideration. Only one aspect, the impact of PPE, was considered while evaluating the adverse events. However, there may have been certain underlying illnesses that could have contributed to the severity of these effects and were not reported during the data collection. Adverse effects were evaluated subjectively and self-reported and could not be verified by the investigator.

Nevertheless, using an online questionnaire was the most reasonable and practical approach to reach out to participants working in different work locations within the hospital. Furthermore, the emotional aspects of wearing PPE are yet to be investigated. The impact of adequate supply and demand of PPE on the mental health and well-being of HCWs should be assessed.

## Conclusions

The adverse effects related to PPE among HCWs should not be underestimated. Solving these issues will ensure the efficiency and quality of work. The availability, safety, and effectiveness of PPE are crucial to help protect HCWs. Studies must examine the quality, characteristics, efficacy, and optimal use of PPE to maintain a healthy workforce capable of caring for patients during the pandemic. The menace of COVID-19 is yet to be contained worldwide, so the findings of this study are significant in the present scenario. Designing effective PPE and educating HCWs on preventive measures are appropriate strategies that should be taken to prevent adverse effects.
